# Metagenomic Analysis of Gut Microbiota for Abdominal Aortic Aneurysm

**DOI:** 10.3400/avd.oa.24-00105

**Published:** 2025-01-07

**Authors:** Eisaku Ito, Takao Ohki, Naoki Toya, Takuo Emoto, Tomoya Yamashita, Tomomi Sugiyama, Takuji Yamada, Hiroshi Mori, Atsushi Toyoda, Ken-Ichi Hirata

**Affiliations:** 1 Division of Vascular Surgery, The Jikei University Kashiwa Hospital, Kashiwa, Chiba, Japan; 2 Division of Vascular Surgery, Department of Surgery, The Jikei University School of Medicine, Tokyo, Japan; 3 Division of Cardiovascular Medicine, Kobe University Graduate School of Medicine, Kobe, Hyogo, Japan; 4 School of Life Science and Technology, Tokyo Institute of Technology, Tokyo, Japan; 5 Advanced Genomics Center, National Institute of Genetics, Mishima, Sizuoka, Japan

**Keywords:** abdominal aortic aneurysm, gastrointestinal microbiome, alpha lipoic acid

## Abstract

**Objectives:** The pathophysiological mechanism of abdominal aortic aneurysm (AAA) remains unclear. We previously reported that *Bifidobacterium adolescentis* levels were reduced in the feces of patients with AAA by 16S ribosomal ribonucleic acid (RNA) gene sequencing. In this study, we increased the number of cases and conducted metagenomic analyses to examine bacterial genes associated with the pathophysiology of AAA.

**Methods:** For gut microbiota data, feces from 55 patients with AAA and 52 patients with no history of AAA, lower extremity artery disease, or coronary artery disease (control group) were collected. Metagenomic analysis was performed by collecting raw stool samples from patients. For intestinal microbiota analysis, metagenomic analysis of the fecal samples was performed.

**Results:** Oral bacteria, including *Actinomyces oris* (*p* <0.0001), *Streptococcus salivarius* (*p* <0.001), *Lactobacillus salivarius* (*p* <0.001), and *Streptococcus* sp. (*p* <0.001), were increased in the feces of patients with AAA. In addition, bacterial genes related to alpha lipoic acid (ALA) biosynthesis (M00882, M00883, and M00884, *p* <0.0001) were decreased in patients with AAA.

**Conclusions:** In the feces of patients with AAA, there was an increase in oral bacteria, and the expression of bacterial genes related to ALA biosynthesis was reduced. The results suggest the possibility of developing gut microbial drug treatments for AAA.

## Introduction

An abdominal aortic aneurysm (AAA) is a fatal condition in which the aorta enlarges and eventually ruptures.^1^^,^^2)^ AAA progresses without symptoms until it ruptures; therefore, it is often discovered during health checkups or examinations for other diseases. Although treatment options include open surgical repair and endovascular aneurysm repair, drug therapy has not yet been established.^3^^–^^6)^ Therefore, if the patient does not meet the requirement for invasive treatment, the patient is subjected to follow-up observation until the diameter of the aneurysm expands. The known risk factors for AAA enlargement include ethnic differences, hypertension, and smoking. However, the mechanism of aneurysm enlargement is unknown, and there is no effective preventive treatment.^7^^–^^9)^

Recently, the relationship between gut microbiota and various diseases has been elucidated.^10^^–^^13)^ In the cardiovascular field, *Bacteroides vulgatus* and *Bacteroides dorei* are associated with arteriosclerosis and heart failure via lipopolysaccharide production.^14)^ We previously reported that the number of *Bifidobacterium adolescentis* was reduced in the feces of patients with AAA.^15)^ However, because the previous study analyzed 16S ribosomal RNA and examined a small number of cases, it was impossible to analyze the association between gut microbiota and the pathophysiology of AAA. In this study, we increased the number of cases and conducted metagenomic analyses to examine the association between bacterial genes and the pathophysiology of AAA.

## Materials and Methods

Data supporting the findings of this study are available from the corresponding author upon reasonable request. The sequence data were deposited with links to the BioProject accession No. PRJDB16790 which was obtained from the DNA Data Bank of the Japan BioProject database (http://trace.ddbj.nig.ac.jp/bioproject/index_e.html).

### Recruitment of patients with AAA and controls

Overall, 55 patients with AAA and 52 controls without AAA, lower extremity artery disease (LEAD), or coronary artery disease (CAD) were recruited at the Jikei University Kashiwa Hospital (Chiba, Japan) between February 2019 and May 2021, and at the Kobe University Hospital (Hyogo, Japan) between October 2014 and July 2015. The AAA group included patients with AAA who did not undergo aortic surgery or any endovascular treatment. We excluded patients with connective tissue diseases, inflammatory bowel diseases, and those who had received antibiotics known to be associated with gut microbiota within the past 3 months. The controls were patients without AAA, LEAD, and CAD, confirmed on the medical interview, physical examination, blood test, ankle-brachial index, electro-cardiogram, and CT scan.

### DNA extraction and pyrosequencing

The fecal samples collected from participants were stored at −80°C. For DNA extraction, 0.6 M perchloric acid and a stable isotope-labeled internal standard mixture (Wako, Osaka, Japan) were added to the fecal samples. Each mixture was then vigorously vortexed and shaken for 30 min at 4°C. After centrifugation, the resulting supernatants were passed through a 0.22-µm spin filter to remove particulates. The extracts were separated using liquid chromatography on an Intrada Amino Acid column (100 mm × 3 mm; Imtakt Corporation, Kyoto, Japan). DNA in the fecal samples was then extracted for whole-genome shotgun sequencing at Nihon Gene Research Laboratories, following an established procedure.^16)^

### Taxonomic and functional profiling

Quality control was performed on the shotgun paired-end reads obtained from the samples, and the phylogenetic composition profile was calculated. Low-quality regions were removed using FASTP version 0.20.1,^7^^–^^9)^ and reads derived from PhiX and the human genome were removed using Bowtie2 version 2.2.9.^7^^–^^9)^ In addition, high-quality reads were obtained by removing non-paired-end reads. From these reads, phylogenetic composition profiles were obtained by calculating the relative abundance of genera or species in each sample using the default parameters of MetaPhlAn3 version 3.0.6.^7^^–^^9)^ The HUMAnN3 pipeline^7^^–^^9)^ was used to calculate functional composition profiles. First, high-quality paired-end reads were combined using VSEARCH, version 2.15.2.^7^^–^^9)^ Next, we executed the HUMAnN3 command and used ChocoPhlAn^7^^–^^9)^ to create a table of the relative abundances of genes registered in the Uniref90^7^^–^^9)^ database. For the obtained Uniref90, UniProtKB was annotated using UniProt ID Mapping (https://www.uniprot.org/id-mapping) to obtain the corresponding UniProtID. By annotating KEGG GENES with DIAMOND version 0.9.10,^7^^–^^9)^ we created a UniProt ID-KEGG GENES correspondence table. Using this correspondence table and the KEGG GENES-KO correspondence table (KEGG release 2022-03-07), we obtained the KO relative abundance table by converting UniProt IDs in the relative abundance table into KO names. Furthermore, using the KO-Module correspondence table (KEGG release 2022-03-07), we obtained a module relative abundance table by adding the relative abundances of the KOs that make up each module.

### Statistical analysis

Patient characteristics were analyzed using the chi-square test, unpaired *t*-test, and Mann–Whitney U test. Data are presented as mean ± standard deviation or standard error of the mean for normally distributed data and as median (25–75th percentiles) for non-normally distributed data. Categorical variables were compared between the groups using Fisher’s exact test. We examined the association between the 2 parameters using Spearman’s correlation test. For bioinformatic analyses, taxonomic (genus and species) and Wilcoxon rank-sum tests were performed, and *p*-values were calculated for taxonomic (genus and species), KO, and module profiles. For overall visualization, principal component analysis was performed using taxonomic (genus and species) profiles. Data were analyzed using Stata/IC (STATA Statistical Software, version 14.0; StataCorp LLC, College Station, TX, USA), R software version 4.1.3 (http://www.r-project.org/), R ggplot2 version 3.4.0, Prism version 7.0 (GraphPad Inc., San Diego, CA, USA), and JMP version 10 (SAS Institute, Cary, NC, USA). Statistical significance was set at *p* <0.05.

### Ethical standards

All participants provided written informed consent for enrolment, and the study was conducted in accordance with the guidelines of the Declaration of Helsinki. This study was approved by the Ethics Committee of the Jikei University School of Medicine (approval no. 30-082[9103]) and registered with the University Hospital Medical Information Network Clinical Trials Registry (trial registration no. UMIN000038284).

## Results

### Gut microbial profile in patients with AAA

We enrolled 55 patients with AAA (Jikei, 55 cases) and 52 patients in the control group with no history of AAA, LEAD, or CAD (Kobe, 30 cases; Jikei, 22 cases). Metagenomic analysis of the fecal samples was performed to analyze the intestinal flora. Patient characteristics included age (77 years old vs. 69, *p* <0.001). A significant difference was observed in the use of aspirin (20% vs. 2%, *p* = 0.003). No significant differences were observed in HbA1c, total cholesterol, high-density lipoprotein cholesterol, low-density lipoprotein (LDL) cholesterol, triglyceride, statin use, or proton pump inhibitor (PPI) use. The patient characteristics are shown in **Table 1**.

**Table:** Table 1 Patients characteristics

	Abdominal aortic aneurysm	Control	p-Value
	N (%) or Median (interquartile range)		
Number	55	52	
Age (years)	77 (71−82)	69 (64−74)	<0.001*
Hemoglobin A1c (%)	5.9 (5.6−6.4)	6.0 (5.6−6.2)	0.576*
Total cholesterol (mg/dl)	200 (162−219)	200 (171−221)	0.911*
HDL cholesterol (mg/dl)	51 (42−59)	58 (44−64)	0.067*
LDL cholesterol (mg/dl)	109 (87−136)	118 (91−134)	0.499*
Triglyceride (mg/dl)	134 (108−197)	121 (82−192)	0.240*
Statin use	22 (40%)	15 (29%)	0.225#
Aspirin use	11 (20%)	1 (2%)	0.003#
Proton-pump inhibitor use	22 (40%)	17 (33%)	0.432#

#Chi–square test; *Mann–Whitney U test

HDL: high–density lipoprotein; LDL: low–density lipoprotein

**Figure 1:**
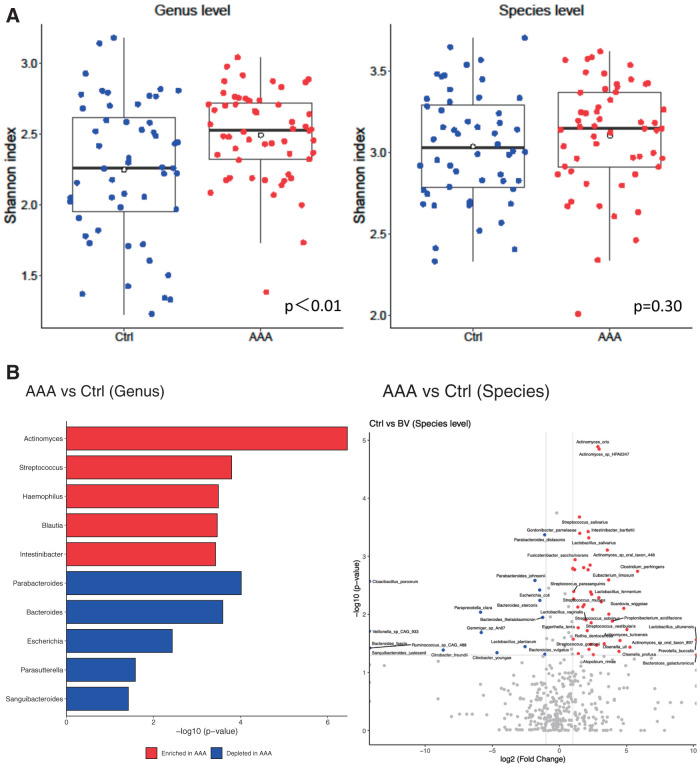
Fig. 1 (**A**) Shannon index for control and abdominal aortic aneurysm. (**B**) Wilcoxon rank sum test for the control and abdominal aortic aneurysm groups.

A comparison of the gut microbiota between the AAA and control groups using the Shannon index and the Wilcoxon rank-sum test revealed differences in the abundance of several bacteria (**Figs. 1A** and **1B**, **Supplementary Fig. 1**). **Table 2** lists the top 10 items that increased and decreased at the genus and species levels, respectively. Oral bacteria such as *Actinomyces oris* (*p* <0.0001), *Streptococcus salivarius* (*p* <0.001), *Lactobacillus salivarius* (*p* <0.001), and *Streptococcus sp*. (*p* <0.001) were increased in the feces of patients with AAA.

**Table:** Table 2 Wilcoxon rank sum test for the control and abdominal aortic aneurysm groups

Genus	
Enriched in abdominal aortic aneurysm	Depleted in abdominal aortic aneurysm
Actinomyces	Escherichia
Streptococcus	Sanguibacteroides
Intestinibacter	Anaeroglobus
Gordonibacter	Acidaminococcus
Lactobacillus	Citrobacter
Fusicatenibacter	Veillonella
Parascardovia	Kosakonia
Scardovia	Bilophila
Parvimonas	Sutterella
Atopobium	Raoutella
Species	
Enriched in abdominal aortic aneurysm	Depleted in abdominal aortic aneurysm
Actinomyces_oris	Haemophilus_parainfluenzae
Actinomyces_sp_HPA0247	Parabacteroides_distasonis
Streptococcus_salivarius	Parabacteroides_iohnsonii
Interinibacter_bartlettii	Cloacibacillus_porcorum
Gordonibacter_pamelaeae	Butyricimonas_synergistica
Lactobacilius_salivarius	Escherichia_coli
Actinomyces_sp_oral_taxon_448	Clostridium_innocuum
Fusicatenibacter_saccharivorans	Bacteroides_stercoris
Parascardovia_denticolens	Clostridium_leptum
Actinomyces_sp_HMSC035G02	Paraprevotella_clara

**Figure 2:**
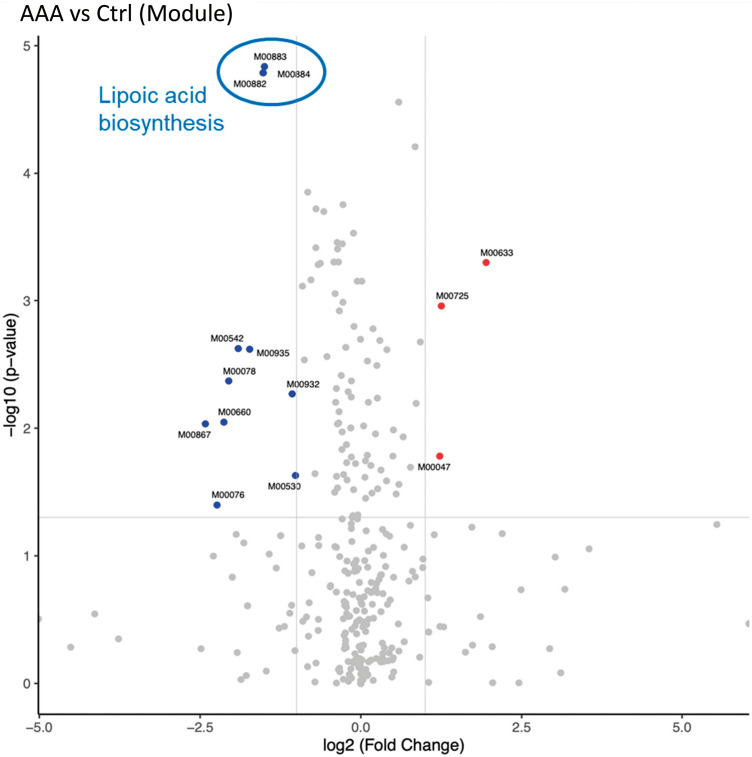
Fig. 2 Volcano plot of KEGG-Module profiles

**Figure 3:**
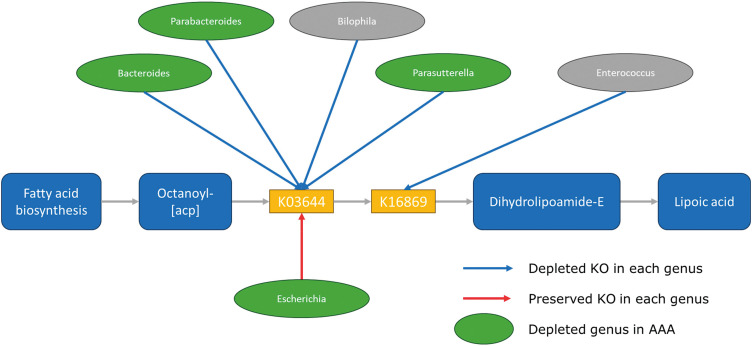
Fig. 3 Lipoic acid biosynthesis: M00882/M00883/M00884. Kyoto Encyclopedia of Genes and Genomes (KEGG) orthology KO genes and their associated genera in Lipoic acid biosynthesis (M00882, M00883, and M00884) are shown. The genera depleted in patients with AAA are indicated by green circles. Arrows are drawn from the genera with differentially enriched KO genes between AAA and controls. The red arrow indicates that the KO genes possessed by each genus were significantly increased in patients with AAA. Blue arrows indicate the KO genes significantly decreased in patients with AAA. AAA: abdominal aortic aneurysm

The volcano plot based on the KEGG-Module profiles showed that the creatine pathway (M00047) increased and the lipoic acid biosynthetic pathway (M00882, M00883, and M00884) decreased in the AAA group (*p* <0.001) (**Fig. 2**, **Table 3**). There was a weak correlation between age and each module (M00882: 0.30, p = 0.026; M00883: 0.29, p = 0.031; M00884: 0.30, p = 0.026). At the genus level, K03644 decreased in *Bacteroides*, *Parabacteroides*, and *Parasutterella* in the AAA group (**Fig. 3**, **Supplementary Fig. 2**). Furthermore, when compared according to the species, K03644 decreased in the presence of the following 4 types of bacteria in the AAA group: *Bacteroides thetaiotaomicron* (*p* <0.001), *Parabacteroides distasonis* (*p* <0.001), *Bacteroides stercoris* (*p* <0.005), and *Bacteroides ovatus* (*p* <0.05) (**Supplementary Fig. 3**). K16869 decreased in *Enterococcus* (*p* <0.05) (**Supplementary Fig. 4**). For multivariate analysis, there was no significant results of the principal component analysis of taxonomic profiles for aneurysm calcification (**Supplementary Fig. 5**).

**Table:** Table 3 Enrichment analysis for module analysis

Name	p-Value
M00883: Lipoic acid biosynthesis, animals and bacteria, octanoyl-ACP => dihydrolipoyl-H => dihydrolipoyl-E2	1.457352e-05
M00882: Lipoic acid biosynthesis, eukaryotes, octanoyl-ACP => dihydrolipoyl-H	1.631766e-05
M00884: Lipoic acid biosynthesis, octanoyl-CoA => dihydrolipoyl-E2	1.631766e-05
M00542: EHEC/EPEC pathogenicity signature, T3SS, and effectors	0.002377206
M00935: Methanofuran biosynthesis	0.002406257
M00078: Heparan sulfate degradation	0.004265605
M00932: Phylloquinone biosynthesis, chorismate (+ phytyl-PP) => phylloquinol	0.005385457
M00660: Xanthomonas spp. pathogenicity signature, T3SS, and effectors	0.008971001
M00867: KDO2-lipid A modification pathway	0.009247458
M00530: Dissimilatory nitrate reduction, nitrate => ammonia	0.02347048

## Discussion

In this study, we found some characteristic changes in the gut microbiota of patients with AAA compared with those of controls without vascular disease. Oral bacteria such as *A. oris*, *S. salivarius*, *L. salivarius*, and *Streptococcus sp*. were increased in the feces of patients with AAA. Additionally, the expression of bacterial genes related to lipoic acid biosynthesis was reduced. The involvement of *Bacteroides thetaiotaomicron*, *Parabacteroides distasonis*, *Bacteroides stercoris*, and *Bacteroides ovatus* was recognized as the cause.

Previous studies have shown that the gut microbiota and oral bacteria are associated with atherosclerotic diseases; however, there are few reports on the relationship between AAA and the gut microbiota.^12^^–^^15^^,^^17^^–^^19)^ When PPIs are taken orally, resident bacteria in the oral cavity, which are usually inactivated by gastric acid, increase in the intestine.^20^^–^^23)^ PPIs are recommended to be prescribed together with antiplatelet drugs to prevent gastric ulcers; therefore, oral bacteria tend to be found in the feces of patients with atherosclerotic diseases.^22)^ Recent reports have shown that an increase in the number of oral cavity-derived *Streptococcus sp*. in the intestine makes worse systemic inflammation and atherosclerotic diseases.^24^^,^^25)^ In this study, there was no significant difference in the rate of PPI administration between the AAA and control groups; however, *Streptococcus sp*. increased in the AAA group. These results suggest that the increase in oral cavity-derived *Streptococcus sp*. may be related to the development of AAA.

Alpha lipoic acid (ALA) is a short-chain fatty acid-containing thiol bond and a cofactor for energy production in the mitochondria.^26)^ It is known to have antioxidant, anti-inflammatory, metal-chelating, and intracellular glutathione-increasing effects.^26)^ ALA improves mitochondrial dysfunction and promotes nitric oxide synthesis in human aortic endothelial cells.^27)^ In a mouse model of atherosclerosis, ALA reduced aortic plaque and lipid deposition, aortic wall thickness, and elastic fiber fragmentation and destruction.^28)^ Furthermore, in the Angiotensin II-induced AAA model in APOE-KO mice, serum LDL and Angiotensin II levels were reduced, and AAA formation was suppressed by ALA intake.^29)^ Based on these results, ALA is expected to be a potential novel therapeutic agent for the treatment of AAA. Various factors are intricately associated with the pathophysiology of AAA, including vascular smooth muscle cells, inflammatory cells, matrix metalloproteinases, and extracellular matrix.^4^^,^^30)^ ALA may suppress AAA enlargement by reducing vascular smooth muscle cell apoptosis and improving extracellular matrix degradation.^29)^

### Limitations

This study has some limitations. First, because the cases were limited to Japanese individuals, the generalizability of the results to other ethnicities should be verified. In addition, since we were unable to examine the relationship between the aneurysm diameter expansion speed and ALA, a prospective investigation should be conducted to verify this relationship. In addition, the present results are correlated and do not show a causal relationship; therefore, verification through basic research, including animal experiments, is required.

## Conclusions

In this study, some characteristics were observed in the gut microbiota of patients with AAA; increased oral bacteria and reduced expression of bacterial genes are related to ALA biosynthesis. The results suggest the possibility of developing gut microbial drug treatments for AAA.

## Declarations

### Informed consent

Participants provided written informed consent for enrolment in the study.

### Acknowledgments

We thank Editage (www.editage.jp) for English language editing. This work was supported by the Japan Society for the Promotion of Science KAKENHI grant Nos. 19K18194 (EI), the Japan Heart Foundation Research Grant (EI), and a grant from the Japan Arteriosclerosis Prevention Fund (EI). PRIME from the Japan Agency for Medical Research and Development (18069370, TYamashita). Mochida Memorial Foundation for Medical and Pharmaceutical Research (TE). Japan Foundation for Applied Enzymology (TE). The Grant for Basic Research of the Japanese Circulation Society (TE).

### Disclosure statement

Takao Ohki received advisory fees from W. L. Gore and the Boston Scientific Corporation. The other authors have no conflicts of interest or financial ties to disclose.

### Author contributions

Study design: EI, NT, TE, TYS, TS, and TYD

Data collection: EI and NT

Analysis: EI, TO, NT, TE, TYS, TS, TYD, HM, AT, and KH

Investigation: EI

Manuscript preparation: HK and KK

Critical review and revision: all authors

Final approval of the article: all authors

Accountability for all aspects of the work: all authors.

## Supplementary Information

Supplementary Fig. 1 LEfSe analysis for control and abdominal aortic aneurysm

Supplementary Fig. 2 Boxplot analysis for K03644 in each genus

Supplementary Fig. 3 Boxplot analysis for K03644 in each species

Supplementary Fig. 4 Boxplot analysis for K16869 in each genus

Supplementary Fig. 5 Principal component analysis of taxonomic profiles for aneurysm calcification.
