# Supplementary material for: Metagenomic Analysis of Gut Microbiota for Abdominal Aortic Aneurysm
Source: Ann Vasc Dis. 2025 Jan 7;18(1):24-00105. doi: 10.3400/avd.oa.24-00105 (PMC11771153; doi:10.3400/avd.oa.24-00105)

Supplementary Fig. 1
LEfSe analysis for control and abdominal aortic aneurysm

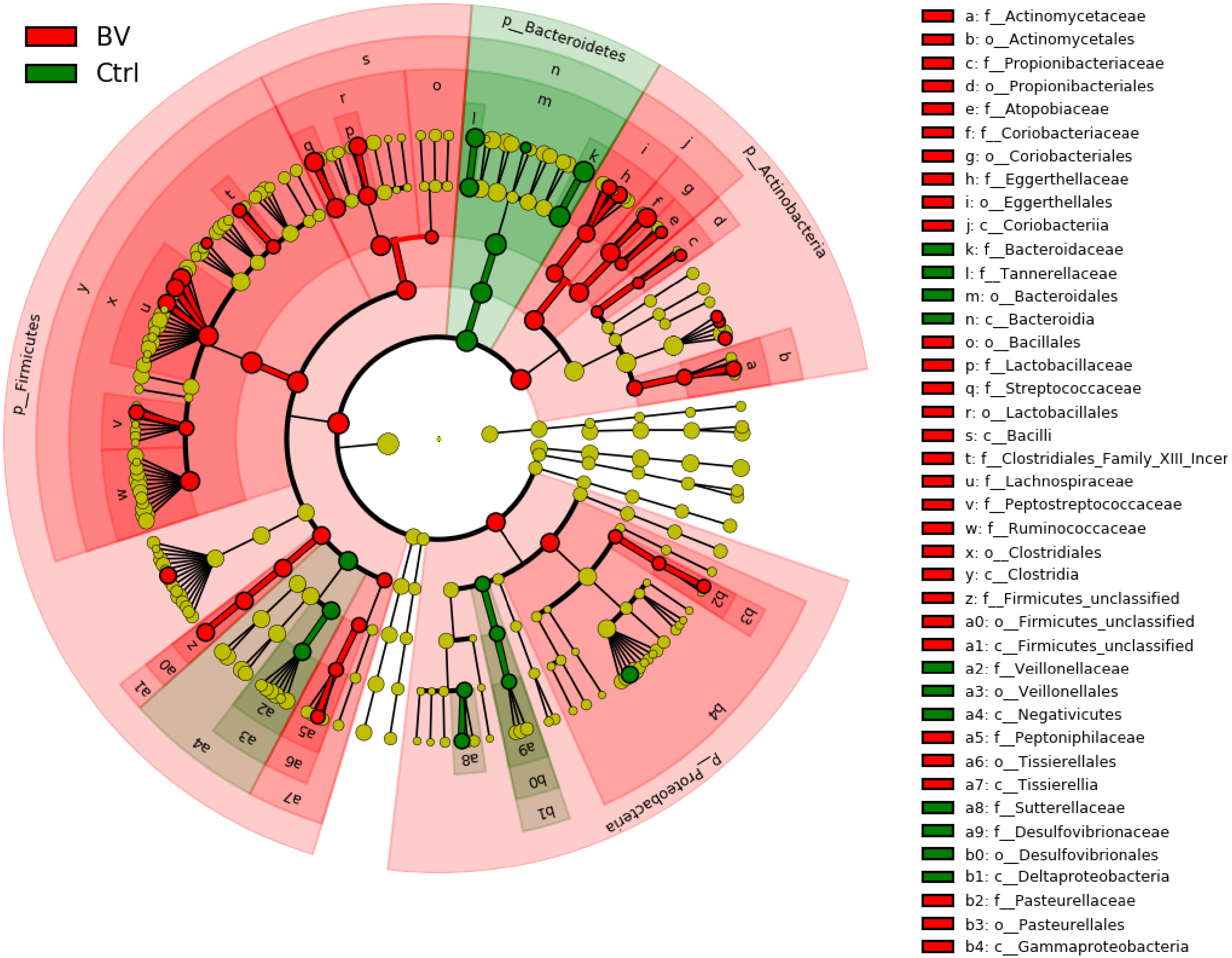

Supplementary Fig. 2
Boxplot analysis for K03644
in each genus

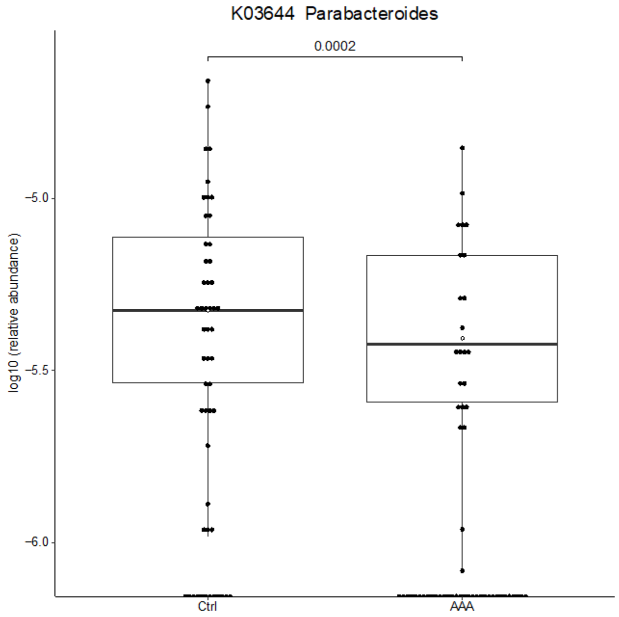

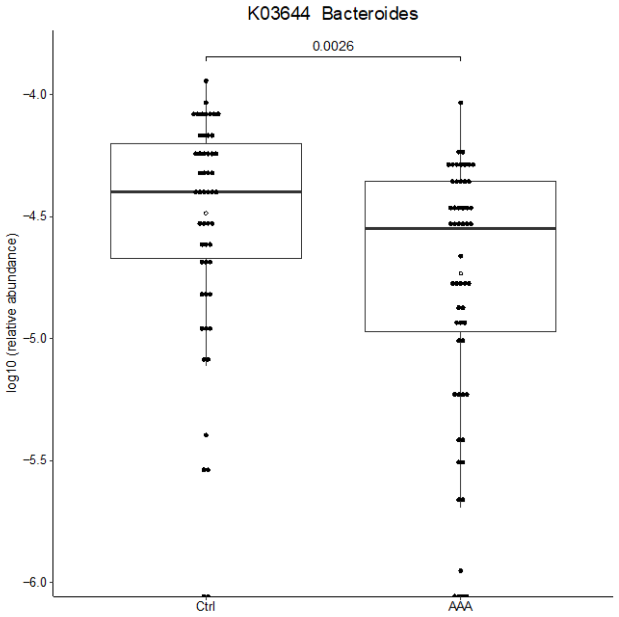

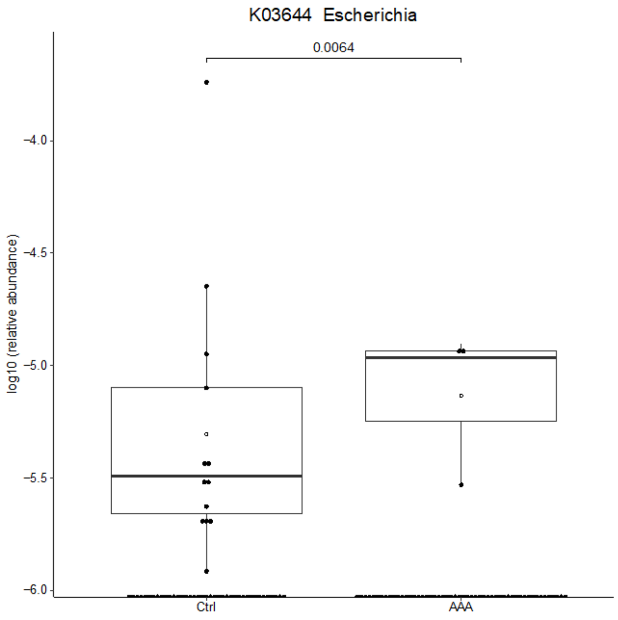

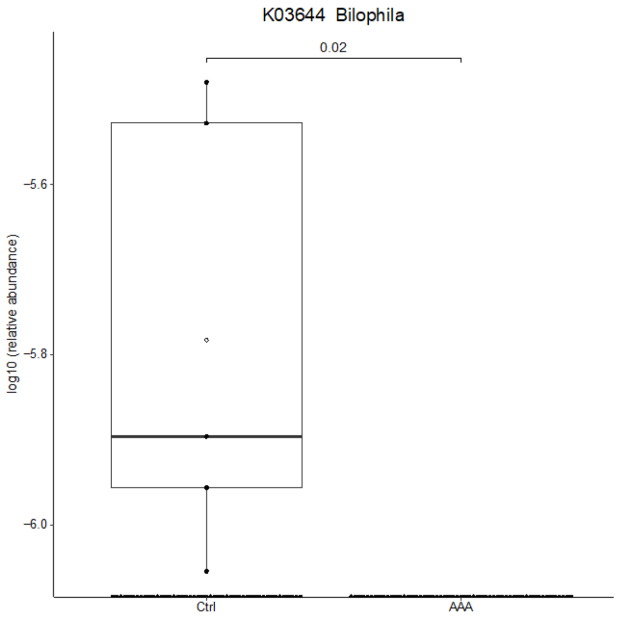

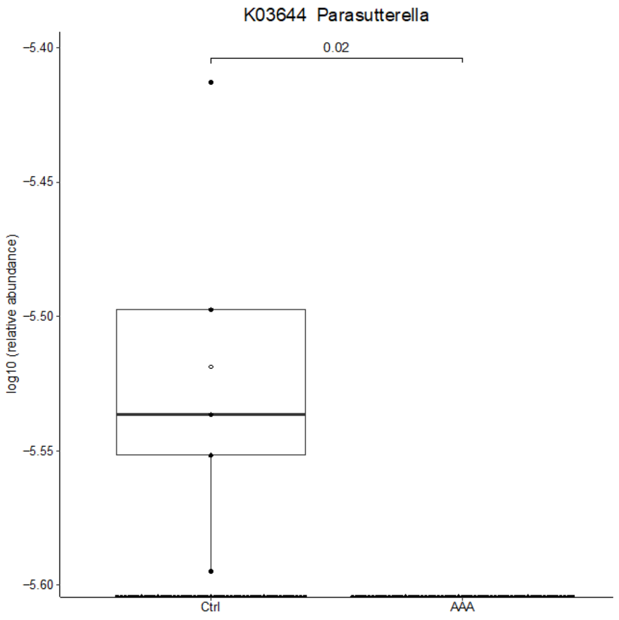

Supplementary Fig. 3
Boxplot analysis for K03644
in each species

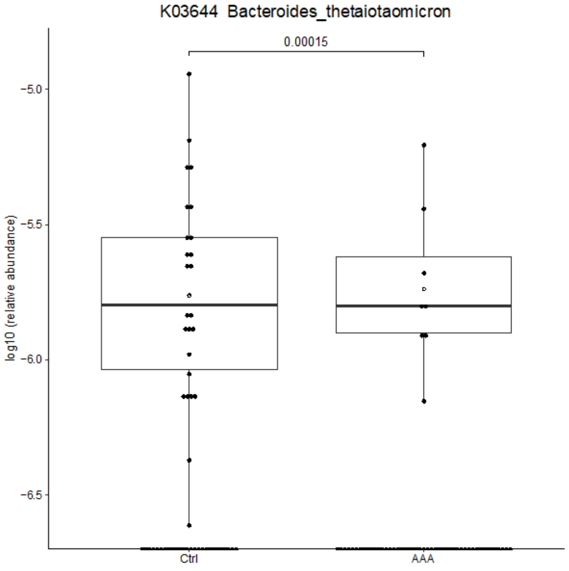

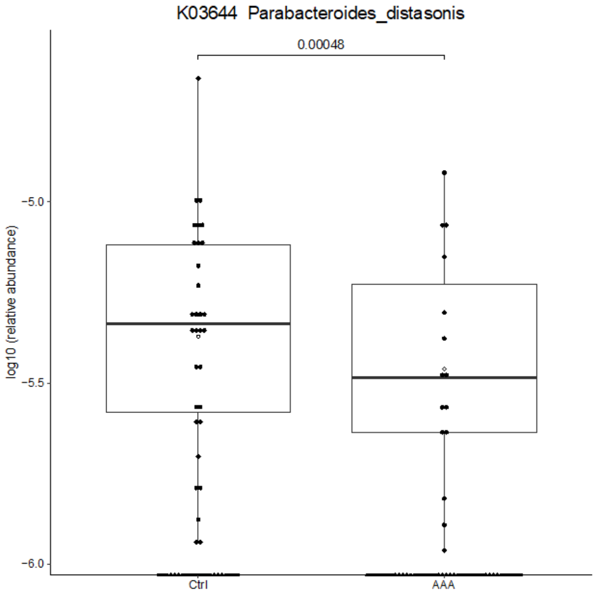

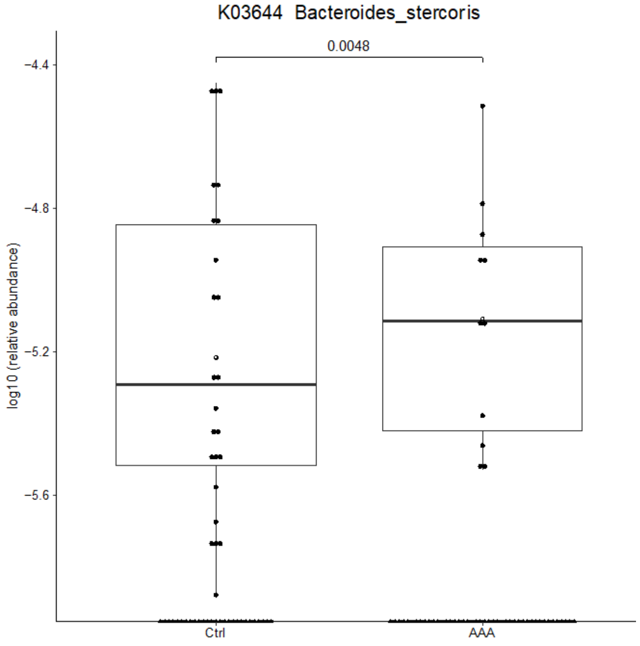

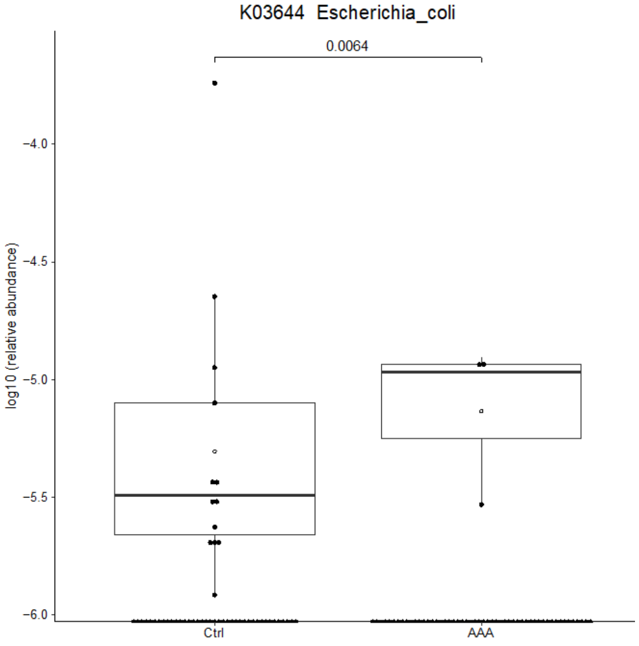

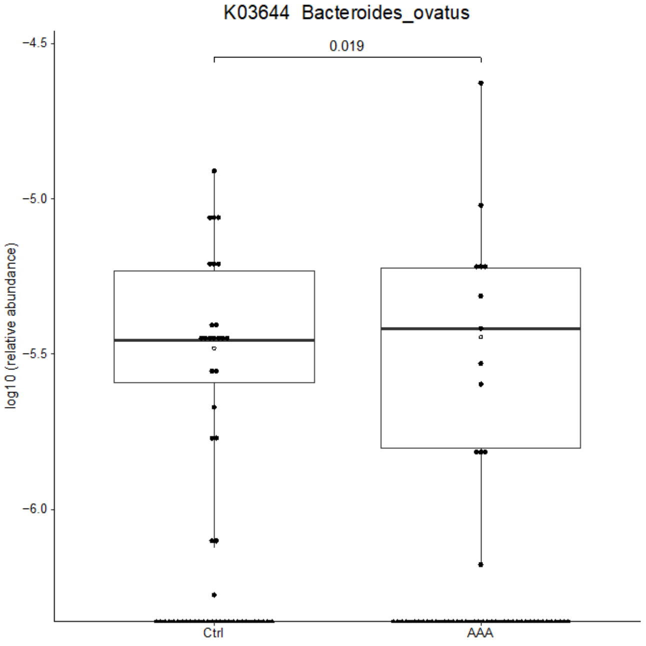

Supplementary Fig. 4
Boxplot analysis for K16869
in each genus

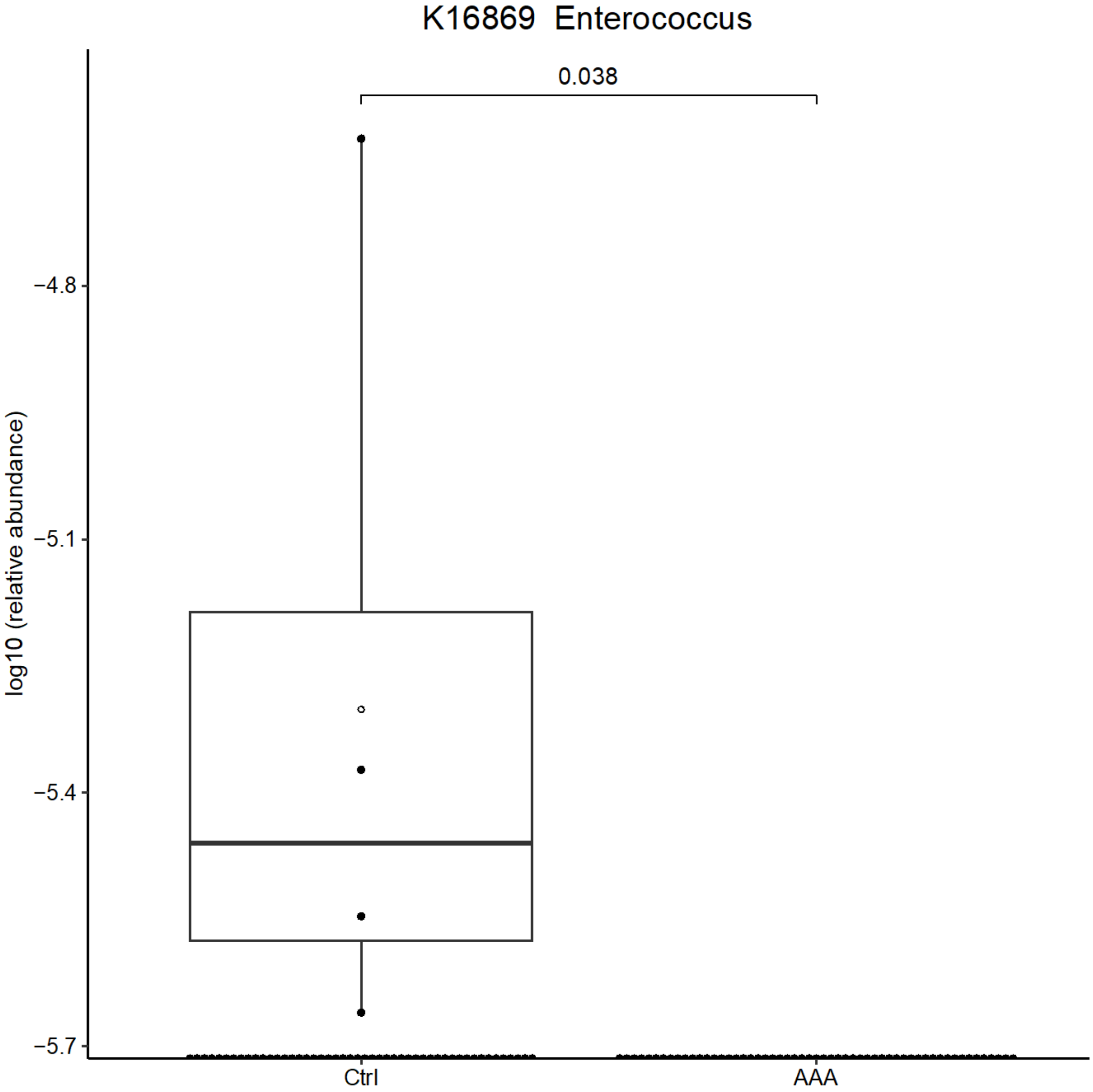

Supplementary Fig. 5

Principal component analysis of taxonomic profiles for aneurysm calcification

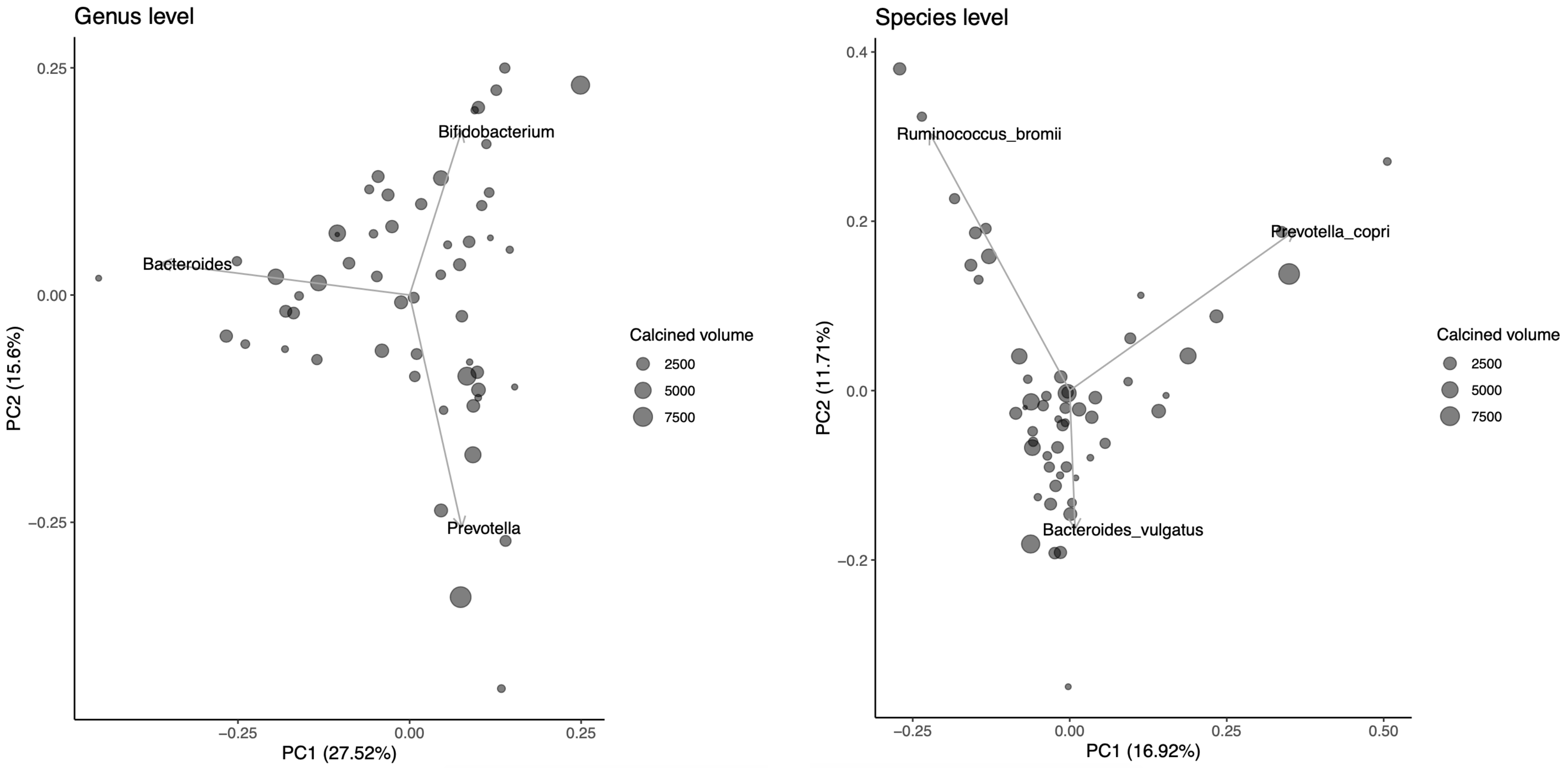

Supplement: Supplementary Fig. 1 — LEfSe analysis for control and abdominal aortic aneurysm [file avd-18-1-24-00105-s01.pdf]
